# Protective Effects of Probiotics on Cognitive and Motor Functions, Anxiety Level, Visceral Sensitivity, Oxidative Stress and Microbiota in Mice with Antibiotic-Induced Dysbiosis

**DOI:** 10.3390/life11080764

**Published:** 2021-07-29

**Authors:** Alisa Arslanova, Aksiniya Tarasova, Anastasia Alexandrova, Vera Novoselova, Ilnar Shaidullov, Dilyara Khusnutdinova, Tatiana Grigoryeva, Dina Yarullina, Olga Yakovleva, Guzel Sitdikova

**Affiliations:** 1 Department of Human and Animal Physiology, Institute of Fundamental Medicine and Biology, Kazan Federal University, 420008 Kazan, Republic of Tatarstan, Russia; AlNArslanova@stud.kpfu.ru (A.A.); AksSTarasova@stud.kpfu.ru (A.T.); IFShajdullov@kpfu.ru (I.S.); olga.jakovleva@kpfu.ru (O.Y.); 2 Department of Microbiology, Institute of Fundamental Medicine and Biology, Kazan Federal University, 420008 Kazan, Republic of Tatarstan, Russia; AYAleksandrova@kpfu.ru (A.A.); VeANovoselova@stud.kpfu.ru (V.N.); Dina.Yarullina@kpfu.ru (D.Y.); 3 “Omics Technologies” Laboratory, Institute of Fundamental Medicine and Biology, Kazan Federal University, 420008 Kazan, Republic of Tatarstan, Russia; DiRHusnutdinova@kpfu.ru (D.K.); 1Tatyana.Grigoreva@kpfu.ru (T.G.)

**Keywords:** antibiotic-induced dysbiosis, microbiota, probiotic lactobacilli, visceral and mechanical sensitivity, locomotor and exploratory activity, muscle strength, motor coordination, anxiety level, cognitive functions, oxidative stress

## Abstract

Accumulating clinical and preclinical data indicate a prominent role of gut microbiota in regulation of physiological functions. The gut-brain axis imbalance due to gut dysbiosis is associated with a range of neurodegenerative diseases. Probiotics were suggested not only to restore intestinal dysbiosis but also modulate stress response and improve mood and anxiety symptoms. In this study, we assessed the effects of probiotic lactobacilli on behavioral reactions, the level of oxidative stress and microbiota content in mice administered to broad-spectrum antibiotics. Our study demonstrates that antibiotic treatment of adolescent mice for two weeks resulted in higher mortality and lower weight gain and induced significant changes in behavior including lower locomotor and exploratory activity, reduced muscle strength, visceral hypersensitivity, higher level of anxiety and impaired cognitive functions compared to the control group. These changes were accompanied by decreased diversity and total amount of bacteria, abundance of *Proteobacteria* and *Verrucomicrobia* phyla, and reduced *Firmicutes/Bacteroides* ratio in the gut microbiota. Moreover, a higher level of oxidative stress was found in brain and skeletal muscle tissues of mice treated with antibiotics. Oral administration of two *Lactobacillus* strains prevented the observed changes and improved not only microbiota content but also the behavioral alterations, suggesting a neuroprotective and antioxidant role of probiotics.

## 1. Introduction

The intestinal microbiota consists of a complex community of microorganisms which inhabit the gastrointestinal ecosystem. Disbalance of this community is able to induce not only various intestinal disorders, but also dysfunctions of the central nervous system, such as impaired cognitive functions [1,2]. Despite the diversity of the intestinal microbiota five types of microorganisms prevail: *Firmicutes, Bacteroidetes, Actinobacteria, Proteobacteria* and *Verrucomicrobia*, including a huge number of classes, genera and species. *Firmicutes* and *Bacteroidetes* account for more than 90% of intestinal microbiota [3,4,5,6]. Although the human-related microbial community is relatively stable, it can change rapidly due to several exogenous and endogenous factors, such as antibiotic therapy, inflammatory diseases, diet or stress [7]. Subsequently, an increased permeability of the intestine and the blood–brain barrier caused by microbial dysbiosis can lead to an invasion of various bacteria and their metabolites from the blood to the brain, resulting in pathological processes in the central nervous system [8]. The role of the gut microbiome in nervous system development, brain functions, and neurodegenerative diseases attracts increasing worldwide research attention giving rise to the concept of the ”gut-brain axis” [2,9,10,11,12].

Recent studies have examined the role of gut microbial imbalances (also called gut dysbiosis) in neurological and psychiatric disorders [13,14]. A more diverse gut microbiome is generally considered healthy (in the absence of elevated pathogenic bacteria) and is associated with improved learning/memory and behavioral flexibility, whereas low microbial diversity is associated with cognitive impairment [15]. Intestinal infections in humans are often associated with increased risk of developing anxiety symptoms over the next two years [16], and healthy mice infected with pathogens have been shown to rapidly display increased anxiety-like behavior [17], even in the absence of a detectable immune response [18], suggesting these microorganisms can directly interact with neural processing.

Antibiotic treatment is the common way to induce dysbiosis, which is characterized by four aspects: loss of key taxa, loss of diversity, changes in metabolic activity and development of a pathogenic microflora [19]. Subsequently, this can lead to the development of chronic inflammation, which is usually associated with higher energy consumption and a predisposition to growth and development disorders [20,21]. Gut–brain axis imbalance caused by antibiotic may result in neurobehavioral alteration the degree of which depends on the type of antibiotic, duration of treatment and the age of animals.

The most common method for dysbiosis treatment is probiotic administration. The beneficial effects are mediated by production of enzymes, promoting fermentation, such as short chain fatty acids (SCFAs), and other metabolites or vitamins [6]. Moreover, recent data suggest beneficial effect of probiotics on the functions of the central nervous system [22,23]. Indeed, probiotic treatment partially reversed cognitive dysfunctions, anxiety or mechanical allodynia in mice due to dysbiosis induced by antibiotics [24,25,26].

In the present study we assessed the effect of broad-spectrum antibiotic administration alone or together with probiotics in mice, investigating locomotor and exploratory activity, muscle strength, motor coordination, anxiety, cognitive functions, visceral and mechanical sensitivity, and the level of oxidative stress in brain and skeletal muscle tissues. We also analyzed alterations in microbiota content in mice undergoing antibiotic and probiotic treatment.

## 2. Materials and Methods

### 2.1. Animals

Experiments were carried out on 25-day old male mice weighing 15–20 g in accordance with EU Directive 2010/63/EU for animal experiments and were approved by the Local Ethical committee at Kazan Federal University (protocol No. 8 from 5.05.2015). Animals were housed in polypropylene cages (4–5 per cage) under controlled temperature (22–24 °C), with a 12:12 light/dark cycle (lights on at 07:00) and free access to food and water.

The animals were randomly divided into 3 groups. Mice from “AB” group (n = 25) received intraperitoneally (i.p.) a cocktail of antibiotics with an interval of one day for two weeks. Each mouse from “AB + LB”group (n = 25) at the day of antibiotic injection additionally received orally once a day 1 mL of lactobacilli (LB) suspension with a concentration of 4 × 106 colony forming units (CFU)/mL. The drinking water contained 2 × 10^5^ CFU/mL of a lactobacilli mixture and was changed with an interval of one day. In addition to antibiotics, they received a 1 mL suspension of lactobacilli at the day of injection. The cocktail of antibiotics for i.p. injections was freshly prepared in 0.8% NaCl and contained in mg/mL: neomycin, 5.0; vancomycin, 25.0; amphotericin B, 0.1; ampicillin, 10; metronidazol, 5.0 [27]. Mice from the control group (n = 25) received injections of saline according to the same protocol.

**Figure 1:**
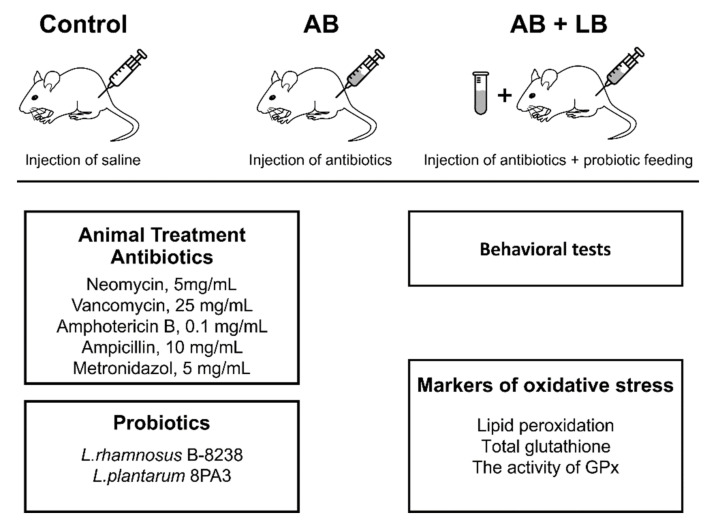
The scheme of the experiments.

The design of experiments is presented in Figure 1. We assessed the mortality and weight of the animals in all experimental groups.

### 2.2. Probiotics

A mixture of two lactobacilli strains was used in our study as probiotics. *Lacticaseibacillus rhamnosus* B-8238 (former *Lactobacillus rhamnosus* B-8238) was obtained from the Russian National Collection of Industrial Microorganisms (VKPM). *Lactiplantibacillus plantarum* 8PA3 (former *Lactobacillus plantarum* 8PA3) was isolated from probiotic preparation “Lactobacterin dry” (Biomed, Vladimir, Russia) as described previously [28]. The bacteria were cultured in de Man, Rogosa, and Sharpe (MRS) broth [29] under microaerophilic conditions at 37 °C for 24 h, harvested by centrifugation at 5000 rpm for 10 min and washed three times with a saline. The washed *L. rhamnosus* B-8238 and *L. plantarum* 8PA3 were separately reconstituted in sterile saline to a final concentration of 4 × 10^6^ CFU/mL, and then were mixed at a ratio of 1:1. Bacteria were freshly prepared with an interval of one day during the two-week experiment.

### 2.3. Microbial Culture Methods for Fecal Microbiota Assessment

Microbiological analyses of mice feces were carried out before the treatment to assess the input parameters of the intestinal microbiota, and on the seventh and fourteenth day of the experiment. A total of 5 g of freshly collected mice feces were thoroughly washed with 10 mL of sterile saline for 30 min at 37 °C and centrifuged at 180–200 rpm. The resulting suspension was serially diluted and plated onto different nutrient agar on Petri dishes as follows. A nonselective nutrient agar for the cultivation of microorganisms “BTN” (Biotechnovacia, Elektrogorsk, Russia) was used as a plate count agar to assess total microbial growth. A cabbage agar with 4% (w/v) CaCO_3_ was used to isolate lactic acid bacteria (LAB). Additionally, we used MRS agar for the cultivation of lactobacilli [29]. Bacteria of the family *Enterobacteriaceae* were inoculated onto Endo agar (Microgen, Moscow, Russia). Plates were incubated at 37 °C for 72 h. To provide anaerobic conditions, we used “Anaerogas” gas-pak sachets (NIKI MLT, St. Ptersburg, Russia). Bacterial colonies were counted and identified based on their morphological properties.

### 2.4. Metagenomic Analyses

#### 2.4.1. Sample Collection

Luminal caecum contents were collected from randomly selected mice of each group. The gastrointestinal tract was removed and caecum was separated. The cecal content (~ 1 g) was placed individually into sterile tubes, thereafter immediately frozen in liquid nitrogen and stored in a freezer at −80  °C.

#### 2.4.2. DNA Extraction

Cecum content samples were collected on the 15th day of the experiment and were stored at −80 °C before homogenization in a SuperFastPrep-1 (MP Biomedicals, Irvine, CA, USA) at 5 m/s for 2 min and DNA was extracted with the Fast DNA SPIN Kit (MP Biomedicals, USA).

#### 2.4.3. 16S rRNA Gene Amplification and Sequencing

Sequences of the V3–V4 variable regions of 16S rRNA gene were analyzed by next-generation sequencing using the MiSeq system (Illumina, San Diego, CA, USA) in 2 × 300 bp mode. A 16S rRNA sequencing library was constructed according to the 16S Metagenomics Sequencing Library Preparation Protocol [30]. Sequencing was performed at the Interdisciplinary Center of Shared Facilities, Kazan Federal University (Kazan, Republic of Tatarstan, Russia) [31].

#### 2.4.4. Quantitative Analysis of Microbiome Composition

Reads were processed and analyzed with QIIME software version 1.9.1 (University of Colorado, Boulder, CO, USA) (http://qiime.org/ accessed on 27 June 2021) [32]. After quality filtering, chimera removal, and rarefaction steps, sequences were clustered into operational taxonomic units (OTUs) at a 97% sequence similarity cutoff. The latest GreenGenes database version 13.8 [33] was used.

To characterize the richness and evenness of the bacterial community, we calculated phylogenetic distance metric (PD_whole_tree), Chao1, Shannon, and Simpson indices. Similarities in bacterial compositions of samples were evaluated using the beta diversity characteristics, and both weighted and unweighted Unifrac were calculated on QIIME software (University of Colorado, Boulder, CO, USA) and further visualized by PCoA.

### 2.5. Behavior Tests

#### 2.5.1. Open Field Test

Locomotor activity and the anxiety level were analyzed in the open field, consisting in this case of a round arena of 60 cm diameter divided into 36 squares of 10 cm × 10 cm (Open Science, Moscow, Russia). The animal’s behavior was recorded for 3 min with a video system and analyzed later by an independent investigator. The following parameters were tracked: horizontal and vertical activity, head dips, grooming, latency to leave the center of the open field, defecation score [34]. The open field was cleaned with 70% ethyl alcohol between trials.

Additionally, we evaluated the dynamics of the anxiety-phobic state of mice during the period of injections in the open field assessing the reaction of an animal to a slowly approaching hand of the experimenter—“Integral indicator of anxiety” (IIA) [35]. The reaction of the mouse was assessed according the following score: 0—no reaction; 1—freezing, moving back, vocalization, dropping ears; reactions disappear when the hand is removed; 2—the reaction remains after the removal of the hand. The test was repeated 3 times and the average result of three approaches was taken. A larger score corresponds to a higher anxiety-phobic level.

#### 2.5.2. The Rotarod Test

Motor coordination was assessed using Rotarod (Neurobotix, Moscow, Russia) with a rotation speed of 5 cm/s. Each mouse was subjected to three test sessions separated by 20–30 min breaks. The time to fall off from the rotating rod was measured and the best result was recorded [36].

#### 2.5.3. Paw Grip Endurance Test (PaGE)

Muscle endurance was assessed by the PaGE where the animal was placed on a wire grid, which was turned upside down over the housing cage [37]. The best time spent on the grid before falling from the three trials was used for analysis.

#### 2.5.4. Light–Dark Box Test

Anxiety level was assessed by the time spent in the light chamber and the number of transitions between the compartments of the light–dark box (Shelter, Open Science, Moscow, Russia) [38]. The animal was allowed to investigate the light–dark box for 3 min. Rodents prefer the dark area but strive to explore new surroundings at the same time. Therefore, this apparatus creates a conflicting situation which results in anxiety-like symptoms.

#### 2.5.5. The New Object Recognition Test (NOR)

The new object recognition test was used to analyze the nonspatial memory of rodents, which is based on the natural tendency of animals to explore new objects and does not require external motivation, reward or punishment [39,40]. The test consists of two 15 min sessions conducted in a home cage. The first session was carried out to test how the mouse adapts and memorizes two identical objects (plastic cubes) placed in opposite corners of the cage. After 15 min, the mouse was removed, one of the familiar objects was replaced with an unfamiliar one (a metal ball). During the second 15 min session, the time (in seconds) spent on the study of new and familiar objects was recorded. The ratio b/a, where “b” is the total exploration time of the novel object and “a” the total exploration time of the familiar object, was evaluated as NOR score.

#### 2.5.6. T Maze

Spatial working memory was assessed in a spontaneous alternation task using a T maze [41] equipped with a video-tracking system (Open Science, Moscow, Russia). Initially, the mouse was allowed to explore the right or left goal arm for 3 min. After the mouse entered into the one of the arms of the maze it was closed for 30 s and then was placed at the starting point (the arm at the bottom of the “T”) before repeating the run. “Alternation” was considered if the mouse entered the opposite arm as compared to the previous run. Each mouse underwent 3 trials separated by 10 min breaks. Alternation in each trial was counted as 33.3% and in the case of selection of the opposite arm in three trials the mouse scored 99.9%.

#### 2.5.7. Von Frey Test

A series of calibrated filaments with strength ranging from 0.008 to 0.6 g of target force, corresponding to 2.53 to 18.4 g/mm^2^ pressure (Ugo Basile, Gemonio, Italy) were used in von Frey tests. In this test, mice were placed individually in small cages with a mesh floor and allowed to acclimatize for 1 h until major grooming and exploration activities ceased. The “ascending stimulus” method was used to estimate the mechanical withdrawal threshold as previously described [42,43]. The von Frey filaments were applied perpendicularly to the plantar surface of the hind paw in an ascending mode with an interval of 10 s between each filament. A response was considered positive if the animal exhibited any nociceptive behaviors, including brisk paw withdrawal, licking, or shaking of the paw, either during application of the stimulus or immediately after the filament was removed. The first filament that evoked at least one response was assigned as the mechanical withdrawal threshold.

#### 2.5.8. Visceral Sensitivity

Mice colon sensitivity was assessed by measuring the threshold intensity of the abdominal withdrawal reflex (AWR) arising in response to colorectal distention in 40–45 days old mice [44]. The distention was applied using 4F-arterial embolectomy catheter (Edwards Lifesciences, Irvine, CA, USA). Measurements of AWR levels were carried out 30 min after wakefulness and adaptation of the animal and consisted of visual observation of reaction to rapid balloon distension for 20 s in ascending order (0.1, 0.25, 0.35, 0.5 and 0.65). The animal’s response to colon dilation was repeated three times with an interval of 30 s and was assessed on the AWR scale: 0, no behavioral response; 1, brief movement of the head, followed by immobility; 2, contraction of abdominal muscles; 3, lifting the abdomen; 4, body flexion and pelvic lift [45,46].

### 2.6. Lipid Peroxidation and the Activity of Glutathione Peroxidases

The brain tissues were homogenized in buffer solution (0.15 M NaCl with phosphate buffer, ratio 1:10) and frozen.

Malondialdehyde (MDA) was measured spectrophotometrically according to the method described by Ohkawa et al. (1979) [47]. Trichloroacetic acid at a concentration of 20% and 0.03 M 2-thiobarbituric acid was added to homogenates of brain tissue at a ratio of 2:2:1. The mixture was heated for 45 min at 95 °C and centrifuged for 10 min at 10,000 *g*. The absorbance of the supernatant was registered at 532 nm by spectrophotometry (PE-5300VI, ECOHIM, Moscow, Russia). MDA levels were expressed as µg/g of tissues.

The antioxidant potential was determined by measuring the activity of glutathione peroxidases (GPx) using tert-butyl hydroperoxide as a substrate according to the method described by Razygraev et al. (2018) [48].

Glutathione solution was mixed with brain homogenate at a ratio of 1:1; then the mixture was divided into two tubes (test and control) and incubated for 5 min. Tert-butyl hydroperoxide solution (5 μM, 0.02 mL) was added to the test tube and incubated for 10 min at 20 °C. After 0.2 mL of cold 10% trichloroacetic acid was added into the test and control tubes, samples were centrifuged for 15 min at 10,000 *g*. A total of 0.1 mL of the supernatant from the control and test tubes was transferred into chemical tubes, and 2 mL of phosphate buffer (pH 8.0) and 0.05 mL of Ellman’s reagent were added and mixed. The absorbance of the control and test samples was measured at 412 nm by spectrophotometry (PE-5300VI, ECOHIM, Russia). GPx activity was expressed as µg/g of tissues per min.

### 2.7. Statistical Analysis

Normality of the sample data was evaluated with the Shapiro–Wilk test (sample size less than 25) for equal variances using *F*-test Origin Pro software (OriginLab Corp, Northampton, MA, USA). Data are expressed as mean ± SEM. Statistical significance between medians was calculated using nonparametric ANOVA Kruskal–Wallis test and Mann–Whitney test in Origin Pro 2015 (OriginLab Corp, USA). Differences were considered as statistically significant at *p* < 0.05; n indicates the number of animals.

## 3. Results

### 3.1. Weight Gain and Mortality

**Figure 2:**
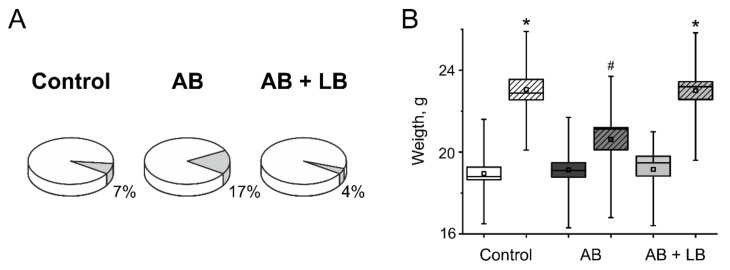
Mortality and weight gain of the mice during administration of antibiotics and lactobacilli treatment. (**A**) Mortality (grey sector) in control, AB and AB + LB groups. (**B**) The weight gain before (unshaded boxes) and after two weeks treatment (shaded boxes) in the control (white); AB (dark grey); AB + LB (light grey) groups. Boxes—SEM, black line—median, the circle inside—mean value, whiskers—5–95 percentiles of dates. * *p* < 0.05 compared to the initial values, # *p* < 0.05 compared to the control group.

The analysis of animal survival during experiments showed the highest mortality rate in AB group of mice (17%, Figure 2A), whereas the control and AB + LB group had a low mortality rate (7% and 4%, respectively).

Moreover, injection of antibiotics results in a lower weight gain of mice (20.6 ± 0.5 g, n = 21) compared to control and AB + LB groups (23.1 ± 0.6 g, n = 23 and 22.9 ± 0.5 g, n = 24, correspondingly, Figure 2B).

### 3.2. Modulation of Gut Microbiota by Antibiotics and Lactobacilli

**Figure 3:**
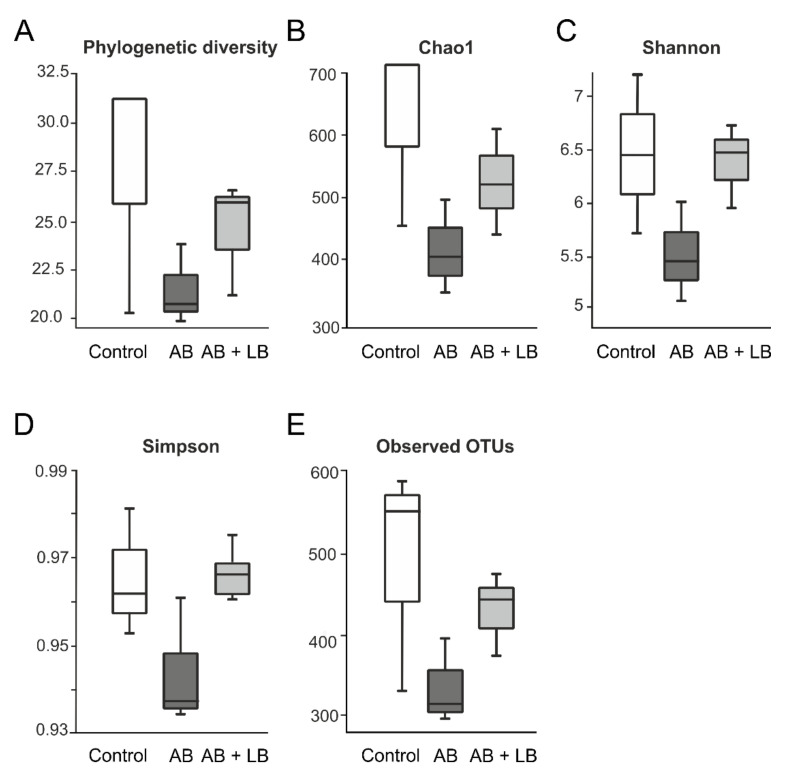
Alpha-diversity box plots. Effects of the broad-spectrum antibiotics alone (AB) and in combination with two *Lactobacillus* strains (AB + LB) on bacterial diversity and richness measures in cecal contents of mice on conclusion of experiments; including phylogenetic diversity (**A**), Chao1 (**B**), Shannon (**C**) and Simpson (**D**) indices, and number of observed bacterial species (**E**). n = 3 per group.

To examine the effects of antibiotics and lactobacilli on the gut microbiota, the bacterial community from cecum samples of each experimental group was analyzed by the conventional culture method and 16S rRNA-based sequencing approach. The alpha-diversity analysis revealed a decreased species richness and evenness in antibiotic-treated mice compared to the control group, but lactobacilli administration alleviated these changes in microbiota content (Figure 3).

**Figure 4:**
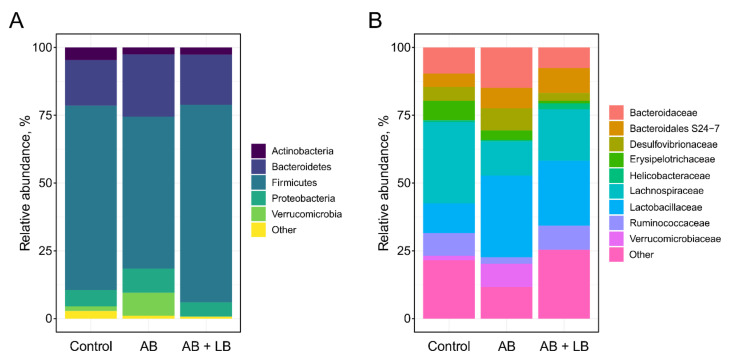
Alterations of cecal microbiota in mice treated with the broad-spectrum antibiotics alone (AB) and in combination with two *Lactobacillus* strains (AB + LB) as compared to the control (Control) group (n = 3 per group). (**A**) Average phylum distribution. (**B**) The most abundant taxa at the family level.

The cecal microbiomes of the mice were predominantly composed of *Firmicutes* and *Bacteroidetes*, followed by *Proteobacteria* and *Actinobacteria*. The treatment with broad-spectrum antibiotics drastically reduced the *Firmicutes/Bacteroidetes* (F/B) ratio, which is widely accepted to be an indicator of dysbiosis [49]. Furthermore, in the AB group we detected the increased abundance of *Proteobacteria* and *Verrucomicrobia* phyla, which are also believed to contribute to dysbiosis [50,51]. The addition of probiotic lactobacilli resulted in the restoration of bacterial phyla content in the gut microbiome (Figure 4A). The families *Bacteroidaceae* (phylum *Bacteroidetes*), *Lachnospiraceae*, *Ruminococcaceae* (phylum *Firmicutes*), *Desulfovibrionaceae* (phylum *Proteobacteria*), and *Verrucomicrobiaceae* (phylum *Verrucomicrobia*) showed similar patterns of population shifts in the family level community analysis as compared with phylum level analysis. When analyzed at the genus level, the most frequently detected taxa in all the samples were *Lactobacillus,* L-*Ruminococcus* (*Ruminococcus* genus assigned to the *Lachnospiraceae* family), and *Bacteroides,* along with unclassified members of the order *Clostridiales* and the family *Bacteroidales* S24-7 (Figure 4B). Surprisingly, we found a significant increase in the population of *Lactobacillus* in both experimental groups as compared to the control. The AB + LB group showed an increase in the abundance of the genus *Oscillospira* and a decrease in the abundance of the genera *Blautia* and *Dorea* as compared with the control or AB group, while the population of unclassified members of the family *Lachnospiraceae* significantly decreased only in the AB group as compared to the other two groups. Additionally, the AB group was largely enriched in the genus *Akkermansia*, whereas in the AB + LB group it was almost absent.

We also compared the gut microbiota profiles obtained by 16S rDNA gene sequencing with those obtained using culture methods (Table 1). As a result, we found discrepancies in the abundance of certain groups of bacteria, depending on the method used. Thus, in contrast to the results of 16S rRNA gene sequencing, increase in the population of lactic acid bacteria (LAB) (order *Lactobacillales*) and more specifically lactobacilli (genus *Lactobacillus*) was not detected by culture method in AB and AB + LB groups as compared to the control. Moreover, the AB group showed a decreased number of lactobacilli, which perhaps resulted from the prevalence of strictly anaerobic *Lactobacillus* spp. that could not be detected by the culture method. Especially drastic discrepancies were obtained by two approaches in regard to the population of *Enterobacteriaceae*. According to the metagenomic studies of 16S rRNA, none of the tested groups had enterobacteria in cecum samples. In contrast, the culture-based technique revealed significant differences in the family *Enterobacteriaceae* between tested groups. Thus, the control group was enriched in lactose-positive enterobacteria, while in AB and AB + LB groups we detected the abundance of lactose-negative enterobacteria, which may indicate a dysbiotic condition in the gut. These controversial results of culture-independent genomic and culture approaches may be attributed to the different kinds of samples used for these analyses. In this work we used metagenomic studies of the 16S rRNA gene for the cecum content samples, while the culture method was applied for fecal samples. When fecal samples of each experimental group were analyzed by the 16S rRNA-based sequencing approach we indeed revealed enterobacteria, which increased in the control group (5.48%) and significantly decreased in AB and AB + LB groups (0.27% and 0.08%, respectively) (unpublished data). Due to known limitations [52], genera of the family *Enterobacteriaceae* with different potential to ferment lactose cannot be differentiated by the genomic approach.

### 3.3. Behavioral Activity Measured in the Open Field Test

**Figure 5:**
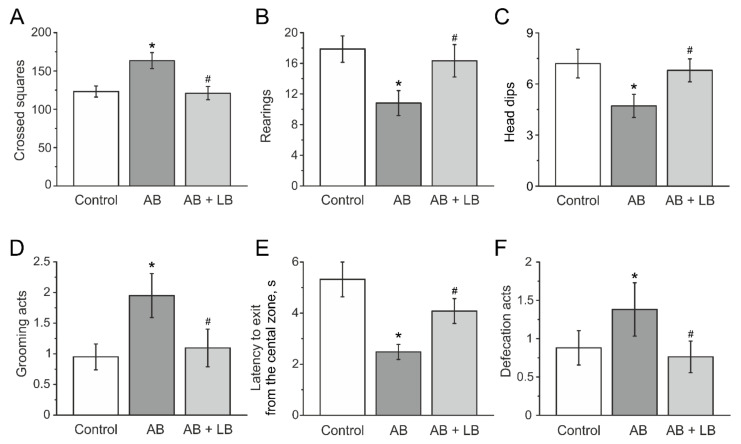
Locomotor and exploratory activity and anxiety of mice with administration of antibiotics and lactobacilli treatment in the open field. Horizontal activity (**A**), rearings (**B**), exploratory activity (**C**), grooming (**D**), latency to exit from the central zone (**E**) and defecation score (**F**) of mice from the control (white columns); AB (grey columns); AB + LB (light grey columns) groups. Data are expressed as mean ± SEM. * *p* < 0.05 compared to the control group, # *p* < 0.05 compared to the AB group.

The initial values of locomotor and exploratory activities and anxiety in the open field were not different in all three groups before the injections and did not change in the control and AB + LB groups after two weeks of injections (Table 2). Injections of antibiotics elevated the number of crossed squares from 128.0 ± 12.4 to 163.5 ± 10.4 in the AB group (n = 21, *p* < 0.05, Figure 5A, Table 2) without changes in the control (123.2 ± 7.2, n = 23) and AB + LB groups (120.4 ± 8.3, n = 24, Figure 5A, Table 2). The number of rearing (vertical activity) in two weeks of injections of antibiotics was significantly lower in the AB mice (10.9 ± 1.7, n = 21, *p* < 0.05) compared to the control (17.8 ± 1.7, n = 23, *p* < 0.05) and AB + LB groups (16.3 ± 2.1, n = 24, Figure 5B, Table 2).

The number of head dips (exploratory activity) decreased from 5.9 ± 0.5 (n = 25) to 4.8 ± 0.7 in the AB group (n = 21, *p* < 0.05, Table 2) and was lower compared to the control and AB + LB groups (7.2 ± 0.8 and 6.8 ± 0.6, n = 23 and n =2 4, respectively, Figure 5C, Table 2).

In the AB group grooming behavior and defecation score were higher (Figure 5D,F, Table 2), whereas the time spent in the center of the open field was lower compared to the control and AB + LB groups, indicating higher anxiety (Figure 5E, Table 2).

### 3.4. Lactobacilli Treatments Decreased the Anxiety Level of Mice with Administration of Antibiotics

**Figure 6:**
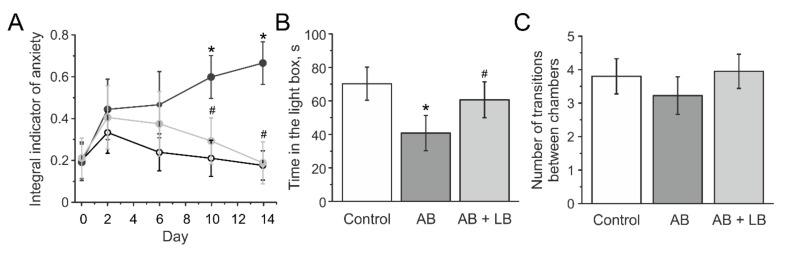
The anxiety level of mice with administration of antibiotics and treatment with lactobacilli. (**A)** Integral anxiety value of mice from control (white circles), AB (dark grey circle) and AB + LB (light grey circle) groups before (0) and during injections of antibiotics for 14 days. (**B**,**C**) Time spent in the light box and number of transitions between the chambers of mice from the control (white columns); AB (grey columns); AB + LB (light grey columns) groups. Data are expressed as mean ± SEM. * *p* < 0.05 compared to the control group.

The dynamics of the anxiety-phobic state before and during injections were measured using the “Integral indicator of anxiety” (IIA) test (Figure 6). The initial value of IIA was not different in all groups: 0.20 ± 0.08 in control, 0.19 ± 0.08 in AB, and 0.21 ± 0.96 in AB + LB groups. After the first injection a local increase in IIA was shown in all groups (Figure 6A), which may be associated with a novel annoying factor. Further, IIA decreased in the control and AB + LB groups indicating habituation of mice to injections. At the same time IIA consistently increased in mice of the AB group and was significantly higher compared to the control and AB + LB groups by the 10th day of injection (Figure 6 A). Mice from the AB group demonstrated a high anxiety level and aggressive behavior by the 14th day of experimentation.

The time spent in the light chamber did not change in the control group before and after injections and was 75.3 ± 11.7 and 70.2 ± 9.8 s (n = 23, *p* > 0.05), respectively. In the AB group this parameter decreased from 73.0 ± 8.1 to 40.8 ± 10.5 s (n = 21; *p* < 0.05, Figure 6B) and was significantly lower compared to the control and AB + LB groups. In AB + LB the time spent in the light compartment did not change before and after injections (77.7 ± 7.8 and 60.6 ± 10.7 s, respectively, n = 24, *p* > 0.05) (Figure 6B). No changes in the number of transitions between the light and dark chambers were observed in all groups (Figure 6C).

### 3.5. Lactobacilli Increases Muscle Endurance and Motor Coordination of Mice with Antibiotic Treatment

**Figure 7:**
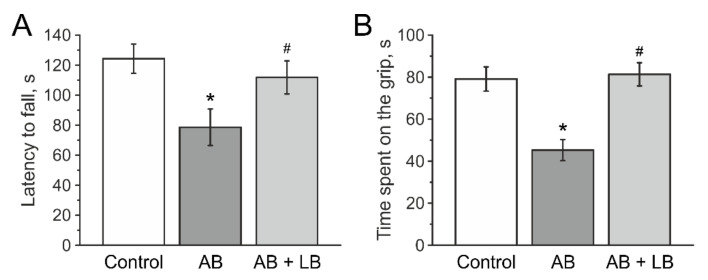
Motor coordination and muscle strength of mice after administration of antibiotics and treatment with lactobacilli. Latency to fall in Rotarod test (**A**) and the time spent on the grip (before falling) in paw grip endurance test (**B**) of mice from the control (white columns); AB (grey columns); AB + LB (light grey columns) groups. Data are expressed as mean ± SEM. * *p* < 0.05 compared to the control group, # *p* < 0.05 compared to the AB group.

In Rotarod tests two weeks after injections mice from the AB group demonstrated a shorter latency to fall from the rotating rod (78.6 ± 12.1 s, n = 21, *p* < 0.05, Table 2) compared to the control group (124.2 ± 9.7 s, n = 23) and AB + LB group (111.7 ± 11.0 s, n = 24) (Figure 7A, Table 2).

In the paw grip endurance (PaGE) test control mice were able to stay on the grid for 73.7 ± 5.7 s (n = 23, Figure 7B, Table 2). In the AB group this time was lower compared to the control group (45.1 ± 5.5 s; n = 21, *p* < 0.05, Table 2). In the AB + LB group this parameter was not different from the control (78.6 ± 6.3 s; n = 24, Figure 7B, Table 2).

### 3.6. Lactobacilli Treatment Improved the Cognitive Functions of Mice after Antibiotic Administration

**Figure 8:**
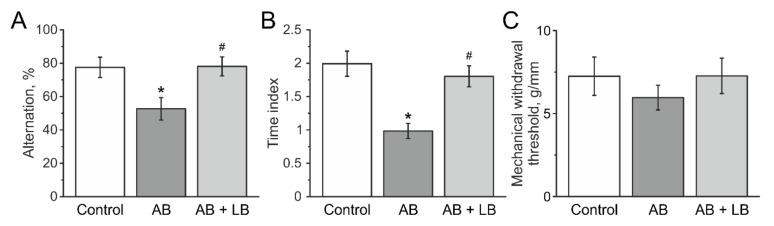
Cognitive dysfunctions and visceral nociception of mice treated with antibiotics and lactobacilli. Percentage of alternation in the T maze (**A**), novel object recognition (NOR) score (**B**) and mechanical withdrawal threshold (**C**) of mice from control (white columns); AB (grey columns); AB + LB (light grey columns) groups. Data are expressed as mean ± SEM. * *p* < 0.05 compared to the control group, # *p* < 0.05 compared to the AB group.

In the T maze test the average percentage of alternation in the control group was 77.4 ± 6.3%, n = 23 (Figure 8A, Table 2). Mice from the AB group showed less alternations (52.8 ± 7.1% (n = 21, *p* < 0.05)) and results obtained from the AB + LB group were not different from the control group (78.7 ± 6.4%, n = 24, Figure 8A, Table 2).

In the novel object recognition (NOR) test the ratio of the exploration time of a novel object to the time exploration of the familiar object was lower in antibiotic-treated mice (1.2 ± 0.1, n = 21, *p* < 0.05) compared to the control (2.0 ± 0.2, n = 23) and the AB + LB groups (1.7 ± 0.1, n = 24, Figure 8B).

### 3.7. Mechanical Sensitivity and Visceral Nociception

Using von Frey filaments, we have shown that the mechanical withdrawal threshold did not significantly change in animals in all groups (Figure 8C).

Analysis of visceral sensitivity demonstrated that AWR values at the balloon volumes of 0.35 mL and 0.5 mL in the AB group were significantly higher compared to the control and AB + LB groups (Table 3), indicating increased visceral nociceptive reaction in mice with antibiotics injections.

### 3.8. The Level of Oxidative Stress in the Brain and Muscle Tissues of Mice after Administration of Antibiotics and Lactobacilli

In order to estimate the level of the oxidative stress in mice after antibiotic administration, the level of MDA and the activity of GPx were measured in brain and muscle tissues of mice from the control, AB and AB + LB groups.

**Figure 9:**
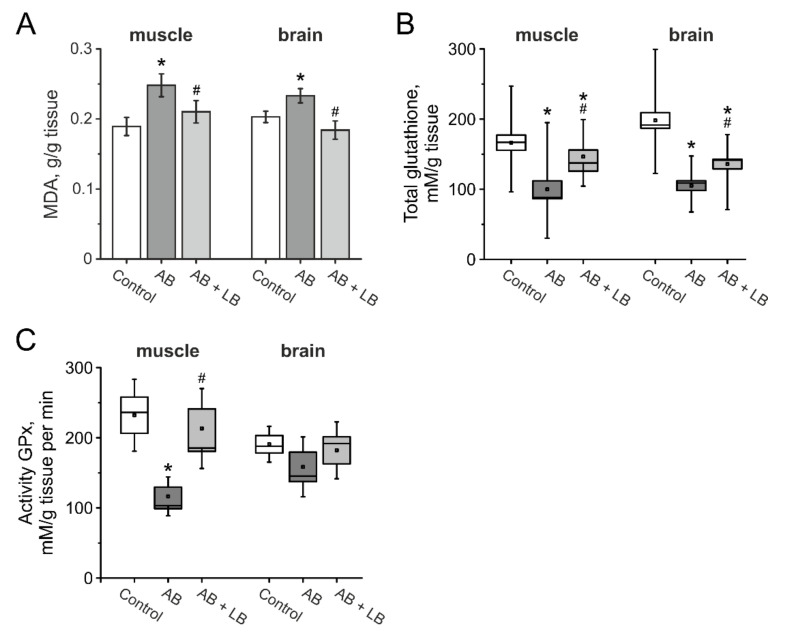
The level of oxidative stress in the brain and skeletal muscle tissue of mice after administration of antibiotics and lactobacilli. MDA level (**A**), total glutathione (**B**), and activity of glutathione peroxidase (**C**) in muscle and brain tissue of mice from the control (white columns); AB (grey columns); AB + LB (light grey columns) groups. Boxes—SEM, black line—median, the circle inside—mean value, whiskers—5–95 percentiles of dates. * *p* < 0.05 compared to the control group, # *p* < 0.05 compared to the AB group.

The MDA level increased in the AB group in muscle and brain, which indicates a higher production of reactive oxygen species (ROS) in mice with antibiotic therapy (Figure 9A). In mice of the AB + LB group the MDA level was significantly lower and did not differ from the control group (Figure 9A). Furthermore, we analyzed the concentration of glutathione and the enzymatic activities of GPx in brain and muscle tissues. We found that the concentration of glutathione decreased in the AB group animals and lactobacillus treatment restored its concentration to control values in both tissues (Figure 9B). The activity of GPx was lower in the muscle, but not in the brain of mice from the AB group and lactobacillus treatment restored its activity to control values (Figure 9C).

## 4. Discussion

The gut microbiota plays a critical role in maintaining the function not only of the gastrointestinal tract but also in the health of the host in general [2,53,54]. An increasing amount of data demonstrate the close relationship between microbiota and brain mediated through the nervous system as well as through the endocrine, immune and metabolic system bidirectionally [26,53]. This bidirectional communication between brain and gut is referred to as the “brain-gut axis”. Although several studies pointed at behavioral alterations in rodents after antibiotic treatment, the results were not consistent and were dependent on the type of antibiotic, administration mode and/or age of animals [24,26,28,55,56]. It was also shown that probiotics can improve behavioral changes of animals treated with antibiotics; however, in most of the studies probiotics were used after antibiotic administration and only partially reversed their negative consequences [24,25,26].

In our study we used wide spectrum antibiotics to induce gut dysbiosis and simultaneous treatment with probiotics aimed to prevent the emerging alterations. Administration of antibiotics in our study was followed by the depletion of the bacterial taxa composition, as well as an imbalance in bacterial communities [57,58,59]. At the same time the total number of microorganisms may even increase, due to the active reproduction of facultative anaerobes of the *Proteobacteria* group [59,60]. The 2-week broad-spectrum antibiotic treatment caused a gut dysbiosis with specific changes in the cecal and fecal microbiota composition. Bacterial diversity and species evenness were decreased in antibiotic-treated animals, in agreement with various authors [60,61,62]. Additionally, this dysbiotic state was characterized by the reduction of the *Firmicutes/Bacteroidetes* (F/B) ratio and increases in *Proteobacteria* and *Verrucomicrobia* phyla. Although theses phyla constitute the main bulk of healthy intestinal microbiota [50], similar changes in their relative abundance have been implicated in various pathophysiological states [49,50,51,63,64]. Culture method substitution of lactose-positive by lactose-negative *Enterobacteriaceae* in the feces of the mice also revealed the dysbiotic state in the gut. Except for the latter, administration of two probiotic lactobacilli strains (*L. rhamnosus* B-8238 and *L. plantarum* 8PA3) under antibiotic treatment conditions resulted in alleviation of the dysbiotic changes in gut microbiota. We expected the lactobacilli administration group to show higher abundance of *Lactobacillus* spp. compared with the other groups. However, in our study cecum content samples and feces of all three groups were similarly enriched in *Lactobacillus* spp. This contrasts with other studies [26,65] that indicated that antibiotics alter the bacterial community, decreasing beneficial bacterial genera such as *Lactobacillus* and *Bifidobacterium*. It should be noted that antibiotic treatment may favor the expansion of antibiotic-resistant bacterial groups. Hence, even similar antibiotic treatment might cause different microbial changes depending upon the doses administered, the basal microbial composition or the environmental conditions. Screening of antibiotic resistance indeed revealed a vast incidence of multidrug resistance among lactobacilli [66,67]. Our data suggest that neuroprotective and antioxidant effects of probiotic administration under conditions of antibiotic-induced dysbiosis are caused by the changes in gut homeostasis rather than an increase in the total amount of lactobacilli.

In our study’s antibiotic administration was started in adolescent mice; therefore, we could observe the weight gain in mice from the control group, whereas mice from the AB group showed lower weight gain due to gut dysbiosis. At the same time treatment with Lactobacilli reversed the weight gain to the control level. Moreover, the mortality of mice from the AB group was higher compared to the control and AB + LB groups. Intestinal microbial communities provide intestinal integrity and absorption of nutrients and perform metabolic and endocrine functions, such as fat storage and/or maintenance of a certain level of leptin and insulin [68]. Moreover, gut microbiota metabolites such as short chain fatty acids (SCFA) and bile acids are important in supporting immune homeostasis both within the gut and all over the body [69]. Dysbiosis due to antibiotic treatment results in increased intestinal permeability followed by chronic systemic inflammation and elevated expression of pro-inflammatory cytokines [25,70,71]. At the same time probiotic treatment protects the integrity of the intestinal barrier, reducing the production of pro-inflammatory cytokins, decreasing myeloperoxidase activity, stabilizing immunological homeostasis and thus reducing the risk of infections and chronic inflammatory reactions [20,21,25,71,72].

Antibiotic injections in our study increased the anxiety level observed in the open field test where mice exhibited a higher level of horizontal activity and grooming, and spent less time in the central zone. These findings were supported by the light–dark test where mice from the AB group spent less time in the light box. Moreover, we tracked the changes in anxiety during two weeks of injections and found that aggressive behavior increased in mice from the AB group, whereas in control and Lactobacilli-treated groups the anxiety level diminished by the 14th day of the experiment. Another study showed that microbiota depletion can even reduce anxiety in mice; however, in that study behavioral tests were performed during long-term antibiotic treatment from P21 to P80 [27]. Similar to our finding, a significant increase in anxiety-like behavior was shown in mice specifically treated with ampicillin during adolescence [56], and probiotics ameliorated anxiety and depressive-like behaviors of antibiotic-treated mice [26,53].

The role of microbiota in supporting cognitive functions has attracted more and more attention in recent studies [2,6]. However, previous studies demonstrated ambiguous results. Antibiotic-induced gut dysbiosis in adult mice impaired novel object recognition, but not spatial memory analyzed in a Barnes maze [24,73]. Early-life dysbiosis did not affect cognitive performance in the Morris water maze [55]. In our study spatial memory was assessed using T maze tests, and mice from the AB group showed significantly lower alternations. This finding is in agreement with recent work that revealed impairments of hippocampal-dependent spatial memory in the Morris tests of antibiotic-treated adult mice [26]. Additionally, in nonspatial memory tests assessed by the ability of a mouse to discriminate between the familiar and novel object, AB group performed worse, similar to previous studies independent from the period of antibiotic administration [27,56,73]. These changes were reversed by administration of probiotics during injections of antibiotics in our study, similar to previous observations [25,26,53].

Administration of antibiotics in our study did not change mechanical sensitivity; however, it induced visceral nociception and this behavior was reversed by probiotic treatment. Similarly, antibiotic treatment during the early or late postnatal period did not change the thermal threshold in the hot plate test [24]; however, it induced visceral pain in adult mice which is a feature of irritable bowel syndrome (IBS) [26,46,55]. IBS is characterized by decreased microbiota diversity and altered proportion of bacterial groups producing SCFAs [46,74]. Probiotic consumption was shown to ameliorate the symptoms of IBS, including abdominal pain in clinical studies [75] and animal model studies [76], due to production of SCFAs and/or the inhibitory neurotransmitter γ-amino butyric acid (GABA) [77,78,79,80].

Furthermore, we found a decrease in muscle endurance and lower motor coordination of mice after antibiotic administration. Previous studies demonstrated ambiguous data concerning the role of microbiota in skeletal muscle functions [24,81,82]. However, muscle fatigability was increased and treadmill endurance capacity was reduced in antibiotic-treated mice [81,82,83]. Our results support the hypothesis that gut bacteria are required for skeletal muscle function, affecting muscle metabolism and fiber phenotype and, consequently, exercise performance [82]. Notably, probiotic treatment completely reversed muscle weakness and motor discoordination in the Rotarod test. One of the possible explanations of positive probiotic action is the production of SCFAs, such as acetic acid, which was shown to recover treadmill endurance capacity and grip strength in mice with altered microbiota [81,83].

Possible mechanisms of the behavioral alteration in dysbiosis include inflammation and stress response through activation of the hypothalamic–pituitary–adrenal axis [26,53]. Antibiotic treatment was shown to reduce the BDNF (brain-derived neurotrophic factor) level in serum and its mRNA expression in the hippocampus, which along with changes in the level of several other hormones and neurotransmitters including oxytocin, vasopressin, acetylcholine, noradrenaline, and serotonergic precursor tryptophan, underlie cognitive dysfunctions and anxiety [24,25,27,56,73]. Moreover, in mice with dysbiosis the spontaneous discharge activity of hippocampal pyramidal neurons was highly depressed and the number of activated microglial cells in the hippocampus was increased [24].

The role of oxidative stress was proposed as a factor which associates with cognitive dysfunctions, anxiety and impaired motor skills [84,85]. Recently, antibiotic-induced cognitive decline and hippocampal neuronal damage were shown to be associated with oxidative stress [25,54]. In our study we found that the level of lipid peroxidation in brain and skeletal muscle tissues was higher in the AB group, which can be explained by insufficiency of intracellular antioxidant compounds scavenging reactive oxygen species or decreased antioxidant enzyme activity [85].

Indeed, we found that the concentration of the total glutathione level was lower both in brain and muscles, and the activity of glutathione peroxidase was significantly decreased in skeletal muscles. At the same time probiotics reversed the level of MDA, GPx and glutathione to the control level, preventing the development of oxidative stress. Our data are supported by recent findings that probiotics decreased inflammation and oxidative stress not only in the gastrointestinal tract, but also in the brain of antibiotic-treated animals and moderately protect hippocampal neuronal damage, thus preventing memory decline [25,54,86].

Probiotics can influence composition and diversity of microbiome and, thus, provoke positive behavioral and neurological changes. Yet, direct action of the probiotic strains on the nervous system cannot be excluded. Specific *Lactobacillus* strains are believed to be psychobiotics, that is they can modulate behavior, affect cognitive brain function, and influence learning and memory processes. The mechanisms involved in probiotic-dependent regulation of the neural processes include production of neurotransmitter, neuromodulators and antioxidants [27,87,88,89]. Moreover, probiotics were shown to change the expression of neurotransmitter receptors in the brain. Indeed, *Lactobacillus*-treated vagotomized animals exhibit altered patterns of GABA receptors, resistance to stress-induced anxiety and depression-related behavior [90]. Further analyses of experimental groups fed with lactobacilli without antibiotic treatment are still needed to validate these observations.

There are several limitations of our study. First, in our model of dysbiosis we used intraperitoneal injections of broad-spectrum antibiotics for the depletion of gut microbiota. This route of antibiotic administration can induce chronical stress, which we did not monitor by the biochemical measurements of hormones, such as corticosterone. To neutralize the effects of injections, however, mice from the control group also received injections of saline and we tracked the level of anxiety-phobic state in all groups during injections. Second, microbiota assessment was performed using a small number of animals per groups (n = 3) and different kinds of samples (cecum and fecal samples). However, it was sufficient to demonstrate that antibiotic treatment caused dysbiosis in mice, but lactobacilli alleviated these changes and we used two different approaches—“culturing” and “sequencing”—which complement each other. More animals in groups are necessary to obtain detailed differences in microbiota content between groups. Third, we did not assess the plasma level of pro- and anti-inflammatory cytokines, which is an important aspect of the protective action of probiotic treatment. Therefore, future studies are necessary to identify the comprehensive mechanisms of microbiota effects on brain functioning.

## 5. Conclusions

Our study demonstrates that administration of broad-spectrum antibiotics in adolescent mice for two weeks resulted in higher mortality and lower weight gain and induced significant changes in behavior of animals including decreased locomotor and exploratory activity, reduced muscle strength, visceral hypersensitivity, increased anxiety and impaired cognitive functions. These changes were accompanied by a decreased diversity of gut microbiota and an increase in pathogenic bacteria. Moreover, dysbiosis was associated with a higher level of oxidative stress found in brain and skeletal muscle tissues. At the same time treatment with lactobacilli prevented the observed changes and improved not only microbiota content but also the behavioral alterations. Taken together probiotics have significant preventive and therapeutic potential for restoring a disturbed microflora, but also for supporting cognitive functions, motor behavior and muscle strength as well as prevention of oxidative stress and visceral hypersensitivity.

## Figures and Tables

**Table 1:** The quantitative content of bacteria (lg CFU/g) in the feces of mice assessed by culture method.

	Control	AB	AB + LB
**Total bacterial growth**			
Input data	15.1 ± 0.1	15.6 ± 0.1	15.4 ± 0.1
1 week	15.1 ± 0.1	15.6 ± 0.1	15.0 ± 0.1
2 weeks	15.2 ± 0.1	15.3 ± 0.1	15.8 ± 0.1
**Lactic acid bacteria**			
Input data	15.1 ± 0.1	13.4 ± 0.2	13.5 ± 0.2
1 week	15.1 ± 0.2	15.2 ± 0.3	15.4 ± 0.1
2 weeks	15.7 ± 0.1	15.6 ± 0.1	15.6 ± 0.1
***Lactobacillus*** **spp.**			
Input data	15.1 ± 0.1	15.7 ± 0.1	15.6 ± 0.1
1 week	15.1 ± 0.1	15.3 ± 0.1	15.0 ± 0.1
2 weeks	15.8 ± 0.0	15.4 ± 0.1	15.7 ± 0.1
**Lactose-positive** ***Enterobacteriaceae***			
Input data	14.6 ± 0.1	14.0 ± 0.2	13.6 ± 0.3
1 week	15.4 ± 0.1	14.3 ± 0.3	14.9 ± 0.1
2 weeks	15.0 ± 0.0	0.0 ± 0.0	0.0 ± 0.0
**Lactose-negative** ***Enterobacteriaceae***			
Input data	14.2 ± 0.2	14.5 ± 0.1	14.4 ± 0.1
1 week	13.4 ± 0.1	15.7 ± 0.1	14.1 ± 0.1
2 weeks	0.0 ± 0.0	15.1 ± 0.1	15.6 ± 0.1

Data are shown as mean ± SD.

**Table 2:** Indicators obtained in behavioral tests before and after treatment in control, AB and AB + LB groups.

Parameters	Before Injections	After Injections	Before Injections	After Injections	Before Injections	After Injections
	Control Group n = 25	Control Group n = 23	AB Group n = 25	AB Group n = 21	AB + LB Group n = 25	AB + LB Group n = 24
**Test Open field**						
Crossed squares, number	130.5 ± 13.9	123.2 ± 7.2	128.0 ± 12.4	163.5 ± 10.4 ^&^*	128.5 ± 10.0	120.4 ± 8.3 ^#^
Rearing, number	13.4 ± 1.8	17.8 ± 1.7 ^&^	13.5 ± 2.5	10.9 ± 1.7 ^&^*	12.9 ± 2.2	16.3 ± 2.1 ^&#^
Head dips, number	4.1 ± 0.9	7.2 ± 0.8 ^&^	5.9 ± 0.5	4.8 ± 0.7 *	4.4 ± 0.7	6.8 ± 0.6 ^&#^
Grooming act, number	1.0 ± 0.2	1.0 ± 0.2	0.9 ± 0.2	2.19 ± 0.4 ^&^*	1.1 ± 0.2	1.1 ± 0.3 ^#^
Defecation act, number	0.9 ± 0.4	0.8 ± 0.2	0.9 ± 0.2	1.4 ± 0.3 ^&^*	0.9 ± 0.3	0.8 ± 0.2 ^#^
Latency to exit from central zone, s	4.3 ± 0.4	5.3 ± 0.7	4.3 ± 0.3	2.5 ± 0.3 ^&^*	3.6 ± 0.4	4.1 ± 0.5 ^#^
**Rotarod test**						
Latency to falls, s	80.1 ± 15.4	124.2 ± 9.7 ^&^	82.6 ± 7.9	78.6 ± 12.1 *	82.8 ± 14.5	111.7±11.0 ^&#^
**Paw Grip Endurance**						
Time to spent on the grip, s	80.9 ± 11.9	73.7 ± 5.7	80.0 ± 12.5	45.1 ± 5.5 ^&*^	80.9 ± 12.2	78.6 ± 6.3 ^#^
**T maze**						
Alternation, %	68.0 ± 5.4	77.4 ± 6.3	73.3 ± 6.1	52.8 ± 7.1 ^&^*	73.2 ± 4.8	78.7 ± 6.4 ^#^
**Novel Object Recognition**						
Time index	2.1 ± 0.2	2.0 ± 0.2	2.1 ± 0.9	1.2 ± 0.1 ^&^*	1.9 ± 0.3	1.7 ± 0.1 ^#^

& *p* < 0.05 compared to initial values, * *p* < 0.05 compared to the control group, # *p* < 0.05 compared to the AB group.

**Table 3:** Abdominal withdrawal reflex scores for colon distention.

Group	n	L				
		0.1	0.25	0.35	0.5	0.65
Control	10	0.29 ± 0.11	0.85 ± 0.15	1.77 ± 0.18	2.8 ± 0.09	3.68 ± 0.04
AB	8	0.5 ± 0.07	1.33 ± 0.11	2.67 ± 0.21 *	3.51 ± 0.22 *	3.83 ± 0.16
AB + LB	12	0.33 ± 0.11	0.83 ± 0.16	1.83 ± 0.22 ^#^	2.91 ± 0.12 ^#^	3.67 ± 0.08

* *p* < 0.05 compared to the control group, # *p* < 0.05 compared to the AB group.

## Data Availability

The data presented in this study are available on request from the corresponding author.
